# Spreadability of Metal Powders for Laser-Powder Bed Fusion via Simple Image Processing Steps

**DOI:** 10.3390/ma15010205

**Published:** 2021-12-28

**Authors:** Cekdar Vakifahmetoglu, Beyza Hasdemir, Lisa Biasetto

**Affiliations:** 1 Department of Materials Science and Engineering, Izmir Institute of Technology, Urla, Izmir 35430, Turkey; beyza.hasdemir@sentes.com.tr; 2 Sentes-BIR, R&D Center, Izmir 35730, Turkey; 3 Department of Management and Engineering, University of Padova, Stradella San Nicola 3, 36100 Vicenza, Italy; lisa.biasetto@unipd.it

**Keywords:** metal additive manufacturing, spreadability, powder bed fusion, gas atomization, illumination invariant

## Abstract

This paper investigates the spreadability of the spherical CoCrWMo powder for laser- powder bed fusion (PBF-LB) by using image processing algorithms coded in MATLAB. Besides, it also aims to examine the spreadability dependence with the other characteristics such as powder size distribution, apparent density, angle of repose. Powder blends in four different particle size distributions are prepared, characterized, and spreadability tests are performed with the PBF-LB. The results demonstrate that an increase in fine particle ratio by volume (below 10 µm) enhances the agglomeration and decreases the flowability, causing poor spreadability. These irregularities on the spread layers are quantified with simple illumination invariant analysis. A clear relation between powder spreadability and 3D printed structures properties in terms of residual porosity could not be defined since structural defects in 3D printed parts also depends on other processing parameters such as spatter formation or powder size over layer height ratio.

## 1. Introduction

Multimaterial 3D printing is one of the next evolutionary steps in AM that will further improve the degree of freedom in design. Among 3D printing technologies powder bed fusion (PBF) wherein spread powder layers are consolidated by a heat source to obtain three-dimensional (3D) components, seems to be one of the most promising especially for multimetals 3D printing [1]. However, the use of different metals to build 3D parts in one process, has effects on the whole process chain, starting from the pre-process (CAD design and generation of the printing file, setting of printing parameters) to the building process itself where for instance new delivery devices for the powders must be designed [2].

In PBF processes [3,4,5,6,7,8,9,10] that include selective laser sintering (SLS), selective laser melting (SLM), and electron beam melting (EBM), homogeneous powder spreading is important since a well-controlled layer in terms of thickness and roughness may provide satisfying structural and functional properties. For instance, the poor control of surface roughness in selective laser melted embedded channels for cooling systems may negatively affect the cooling efficiency, in contrast with the optimized design. Homogeneity on the powder bed layer depends on powder properties such as shape, size, and roughness, that affect cohesion and apparent/tapped density, so as impurities (such as the presence of a passivating oxidation layer), moisture, etc. [11,12]. Considering combining different types of powders, the control of spreadability becomes one of the first steps necessary to grant high-quality components.

Standard characterization methods of powder metallurgy cannot unfortunately adequately meet the expected properties for AM. In 2014, the ASTM F42 committee created ASTM F3049 for the characterization of metal powders for AM. Specific powder properties marked are particle size distribution, particle morphology, chemical composition, true density, and impurity content (C, S, O, N, H, etc.). Bulk powder properties are categorized as flow rate, apparent density, tap density, and angle of repose [13]. In this context, the purpose of DIN EN ISO/ASTM 52,907 is to simplify customer and supplied relation to acquire metallic powder for AM [14].

In the past decade, a considerable amount of research has been conducted toward the investigation of powder characteristics for AM [11,12,15,16,17,18,19,20,21,22,23]. Particularly for metal powders used in PBF, a very recent study [15] investigated the effect of spreading velocity, layer thickness, and powder morphology on layer uniformity. In brief, it was shown that texture and sphericity were important to define the spreader velocity which was critically important for the uniformity of the powder bed [15]. Other studies focused on the role of particle-related properties (particle size distribution, morphology, flowability, etc.) on the processability of the Ti6Al4V, AISI 316L, and AlSi10Mg alloys [16,17]. It was shown that, when fine particles (D_50_ = 9 µm) were used because of lower flowability, the components with lower dimensional accuracy were obtained compared to the coarse ones (D_50_ = 40 µm) [17]. Besides, spread characteristics and powder bed packing density were shown to be affected by shape irregularities and particle roughness of the 316L. Bad spreadability can cause inhomogeneity and affect the regularity of the powder bed surface [18]. Additionally, the angle of repose (AOR) is related in many studies to the powder flowability with a brief outcome that is the smaller the AOR the better the flowability [16,19,20]. A different work [21], studied the effect of recycling on the 304 L stainless steel powder for the PBF process. Flowability of the recycled powder was improved due to (i) a decrease in the number of fines, (ii) more spherical, and (iii) surface oxide formation reducing the friction and surface energy at the interparticle contacts lessening adhesion. Similarly, in a recent review on the recycling of metal powders in AM, the importance of flowability on the final component produced by the PBF method was examined [22]. However, still in practical applications, the suitability of the powder layer for PBF is determined by the observation of an operator or by in situ cameras in the 3D printing machine [23]. 

It is not an easy task to form a spread layer with a homogeneous powder distribution which is crucial for achieving the demanded final properties. Spreadability can be defined as a way to quantify the powder distribution through the layer. Currently, there is no standardized characterization method for spreadability for the metal powder layer, and it is currently controlled merely by following the powder flowability, which is the result of the combination of physical properties affecting the flow behavior [24]. In addition to that, there are very limited published works. In 2019, researchers proposed that powder spreadability among all metrics can be determined by the build plate coverage ratio, the rate of powder deposition, and the alteration rate of the avalanche angle in powder-bed systems [20]. In a more recent study [25], it was shown that particle morphology and size distribution was important for a homogenous spread layer and better mechanical properties of the 3D-printed component. Similarly, flowability and apparent density were directly affected by the PSD, surface topology, and morphology of the powder in PBF-LB applications [12]. Moreover, spreading dynamics were investigated by high-speed high-energy X-ray imaging, and it was shown that the average powder size is an important parameter affecting the flow during spreading [26]. In [27], the authors reported an image analysis method to quantify spreadability using a modified thin film applicator machine, with adjustable height and speed doctor blade. Authors showed that the spreadability, quantified by geometrical considerations on the processed image, can be related to the rheometry results (powder specific energy) to avalanche and rest angle.

Few modeling studies have also been conducted to analyze powder spreadability, e.g., Ref. [28] have used the discrete element method (DEM) simulation at the particle level to determine the packing density of the powder layer for the powder-spreading process. The authors concluded that the packing density of powders with a higher portion of fine particles is low due to the agglomeration, i.e., high cohesive forces. The utilization of bimodal (fine and coarse), powder mixtures was not successful as well due possibly to the segregation and voids in the layer. While few studies did similar simulations for powder spreadability characterization as well [29,30,31] no other studies have so far been found to propose a simple technique. Such scarce data are partially due to 3D printer manufacturers since they market their instruments with already defined production parameters and raw metal powder sources (same for polymeric and ceramic materials). The companies impose the utilization of such defined parameters and powder sources to guarantee, and otherwise, they do not provide any assurance of the produced parts. 

This study aims to add knowledge and develop a simple illumination invariant method by investigating the powder characteristics using digital photos acquired by a digital camera and applying an image processing algorithm in MATLAB. Besides, it will be correlated with other existing characterization methods such as flow rate, apparent density, angle of repose of a model CoCrWMo metallic powder which is commonly used in dental applications such as dental crowns and bridges, for PBF-LB. This research may also open new avenues to provide information on the utilization of outsourced powders that can be used in any available machine, a step further study to render a general tool that is applicable in PBF multimaterials processes. 

## 2. Materials and Methods

### 2.1. Powders Bed Analysis 

Spherical CoCrWMo powders (PURESPHERE 43055; Mn:0.08 wt.%, Cr:26.82 wt.%, Mo:4.66 wt.%, W:5.40 wt.%, Si:0.90 wt.%, kindly supplied by Sentes-BIR A.S., Izmir, Turkey) produced by gas atomization were used as-is in all experiments. Four different powder mixtures with different powder size distribution (PSD) were prepared and coded as M15-45, Mix1, Mix2, and Mix3, where M15-45 was the standard PSD of CoCrWMo powders available in the market and optimized for PBF process, while Mix1, Mix2, and Mix3 were sieved to have increasing average powder size. Powders were sieved by a Retsch Sieve Shaker (AS200 Basic, Retsch, Germany) with 20 µm, 38 µm, 45 µm, and 63 µm sieves. The particle size was measured both by laser diffraction analyzer (Malvern Panalytical Mastersizer 2000, Malvern, UK) and by using the SEM images with the ImageJ software (ImageJ 1.52a, National Institutes of Health, USA) to quantify the average data (from 100 measurements). The morphological characterizations of powders were done by scanning electron microscopy (SEM; Thermo Scientific Apreo S, Waltham, MA, USA). All powder mixtures were dried in an oven at 90 °C prior to any spreadability and 3D printing test. 

**Figure 1:**
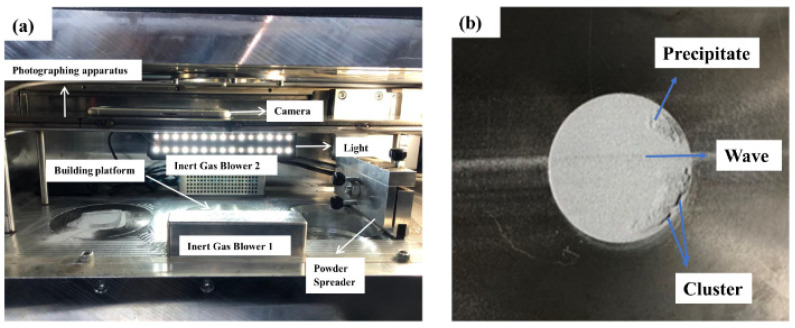
(**a**) Modified Laseral MTL90 Laser-Powder Bed Fusion machine, (**b**) an example of a digital photo taken from 30 mm diameter building platform before applying any MATLAB algorithm. The spread later details can be seen with possible spreading irregularities such as precipitates, clusters, waves.

For spreadability tests, performed at a constant spreading speed (200 mm s^−1^) with a continuous layer thickness of 50 µm, the printer spreading system was modified to install a Bluetooth controlled cell phone fixed with stainless steel apparatus having a 6 mm diameter aperture for the digital camera (8-megapixel, f/2.2, 1.5 µm) of the cell phone, see Figure 1a, for building platform details. A rubber blade on a 30 mm diameter building platform with white LEDs bonded to black cardboard was used.

To characterize the spreadability behavior, preliminary studies were done with the samples having deliberately made irregularities, an example spread layer is shown in Figure 1b. 

**Figure 2:**
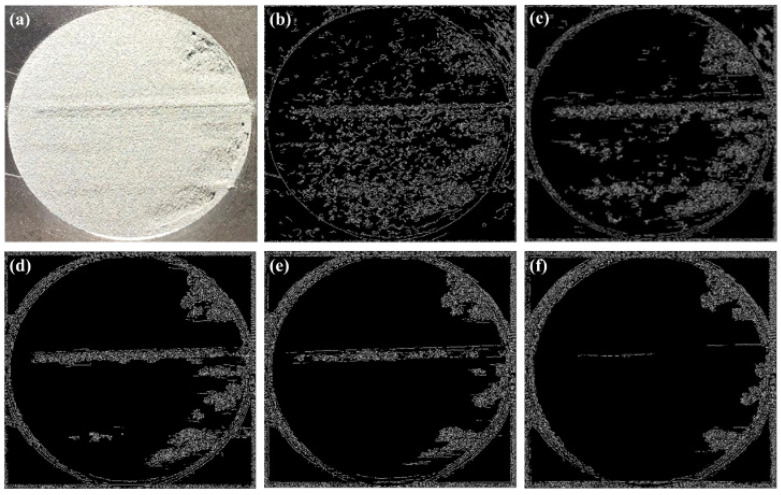
An example poor spread layer images (30 mm circle diameter): (**a**) without any operation, and upon 2D Wiener noise removal filtering with different neighborhood sizes; (**b**) 10, (**c**) 20, (**d**) 30, (**e**) 40, and (**f**) 50.

The camera images of the spreads were processed by Wiener filtering in MATLAB 2019a, (see Figure 2a–f). Initially, an edge detection (“canny”) operation was applied to detect the boundary of the circular region. Then, after finding the center and the radius of the boundary circle using the circular Hough transform, thresholding (Otsu method) was conducted inside the extracted circle to calculate black to white ratio simply from the number of black and white pixels, and further average grayscale value in between 0 and 255. It should be noted that here only average grayscale values are given, however, the standard deviation of grayscale values may be useful to properly demonstrate the homogeneity for which further dedicated studies are needed.

Flow rate (following ASTM 213), apparent density, and angle of repose measurements were conducted by Hall flowmeter (produced according to ASTM B212 standard by Sentes-BIR, Izmir, Turkey) [12]. Tapped density measurement was performed by Tapped Density instrument (produced according to ASTM B527 standard by Sentes-BIR, Izmir, Turkey). The chemical composition of the used powder was measured by inductively coupled plasma emission spectroscopy (Perkin Elmer Avio 200, Waltham, MA, USA). The oxygen amount was measured with an inert gas fusion principle (Leco TC400, LECO, St. Joseph, MI, USA).

### 2.2. 3D Printing of the Powder Bed

Following the image analyses, two cubes for each powder mixture (with an edge length of 8 mm) were produced by 3D printing, followed by sample removal from the platform via wire erosion. PBF-LB (Laseral MTL90 Izmir, Turkey) machine, designed for dental restorations, was used for 3D printing by SLM for which the build chamber environment was kept under pure N_2_ (>99.9% pure) to ensure the oxygen content was limited to 0.1 wt.%. The printing parameters are given in Table 1.

Microstructural investigations, performed by SEM analyses (as reported above), of used powders demonstrated no surface porosity, besides no internal porosity was observed when both fractured bead pieces and Bakelite mounted, ground, and polished samples were examined. The surface porosity of the 3D printed cubes was counted by using a Nikon (Eclipse LV150N, Tokyo, Japan) optical microscope equipped with NIS-Elements BR image analysis. The bulk density measurements were conducted both by Archimedes (following ASTM C20-00 with DI water as buoyancy fluid) and by geometric measurements (for all analyses Radwag AS 220R2 scale was used). The true density was measured by pycnometer (AccuPyc II, Micromeritics, Norcross, GA, USA) according to the ASTM B923-16 standard. 

## 3. Results and Discussion

### 3.1. CoCrWMo Powders Characteristics

The characteristics of the CoCrWMo powder mixtures, coded as M15-45, Mix1, Mix2, and Mix3 analyzed by laser diffraction are given in Table 2. While D10, D50, and D90 values of the M15-45 and Mix2 were similar to each other, M15-45 was used since it is a generally recommended powder distribution type for AM, and still there were slight differences in the amount of the particles below 20 µm and above 45 µm. Mix1 had a high ratio of particles below 20 µm being 66.58%, and instead, Mix3 included coarser particles higher in volume.

**Figure 3:**
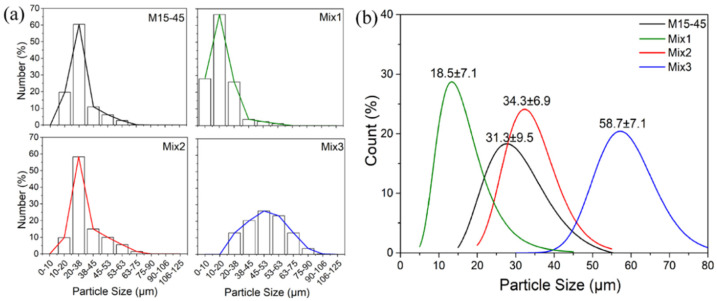
The particle size distribution (PSD) plots of the samples were obtained from; (**a**) laser diffraction, and (**b**) SEM image analysis from 100 measurements.

The particle size distribution (PSD) plots obtained from both laser diffraction and microscopic evaluations (SEM) are given in Figure 3a,b, respectively. For the morphological investigation, the stereological equation D_sphere_ = D_circle_/0.785 was applied to convert 2D measurements to 3D values [32,33]. The average particle size was assessed as; 31.27 ± 9.46 µm for M15-45, 18.54 ± 7.05 µm for Mix1, 34.30 ± 6.97 µm for Mix2, and 58.72 ± 7.08 µm for Mix3, comparable with the laser diffraction obtained D50 values, as seen also in other works [34].

**Figure 4:**
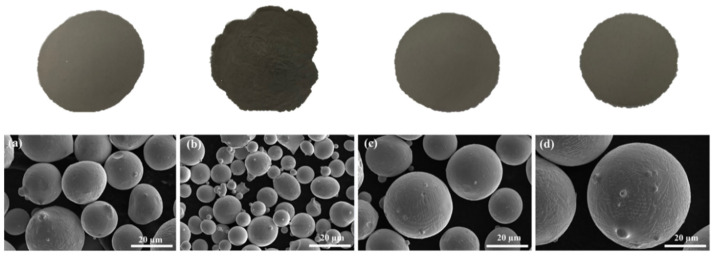
Visual examinations and higher magnification SEM images of: (**a**) M15-45, (**b**) Mix1, (**c**) Mix2, and (**d**) Mix3.

According to visual examinations, M15-45, Mix2, and Mix3 were more flowable, while Mix1 was less flowable due probably to the higher portion of agglomerates, see Figure 4 top. The morphologies of the powder mixtures principally being spherical are shown in Figure 4 bottom. Few non-spherical particles, i.e., irregularities, can be explained with gas atomization process parameters. As known for example the powder morphology is directly related to the cooling rate during atomization that is affected by melting temperature of the metal or alloy, atomization pressure, free-fall distance, and gas to metal ratio. 

The angle of repose (AOR, α) can be calculated via (1) in which H is the height, D is the diameter of the formed powder cone as given in (Equation (1)):

(1) $$\alpha =\tan^{-1}\left(\frac{2H}{D}\right)$$

**Figure 5:**
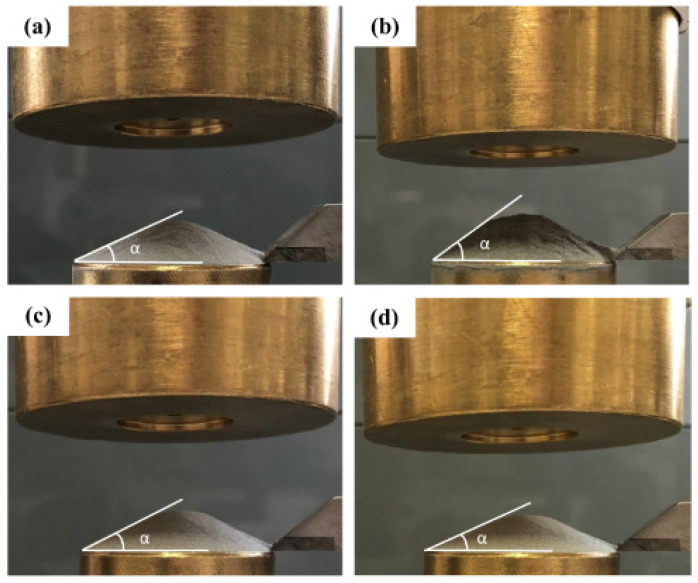
The angle of repose (AOR) measurements of; (**a**) M15-45 (22.39°), (**b**) Mix1 (32.85°), (**c**) Mix2 (22.01°), and (**d**) Mix3 (21.33°).

Figure 5 shows the AOR measurements of all powder mixes. As can be seen, Mix1 was quite cohesive due to the higher fine particle ratio (particles below 20 µm was 66.58 vol%) and was given as 32.85°. Instead, Mix3 was more flowable (particles below 20 µm is 0 vol%) with an AOR value of 21.33°, i.e., as expected a lower flow resulted in a lower AOR [20].

Density and flow rate results are shown in Table 3. The flow rate of Mix1 was not able to be quantified due to high fine powder volume. The lowest flow rate and AOR values were measured as 14.19 (s/50 g) and 21.33 (s/50 g) for Mix3, respectively. The highest apparent density measured was 4.97 g cm^−3^ from Mix1, and the lowest apparent density was measured as 4.78 g cm^−3^ from Mix15-45. As could be seen the highest oxygen content was measured for the Mix1 sample due probably to the higher fine power ratio making the surface area value relatively higher than that of the others. 

### 3.2. Powders Spreadability by Image Processing

**Figure 6:**
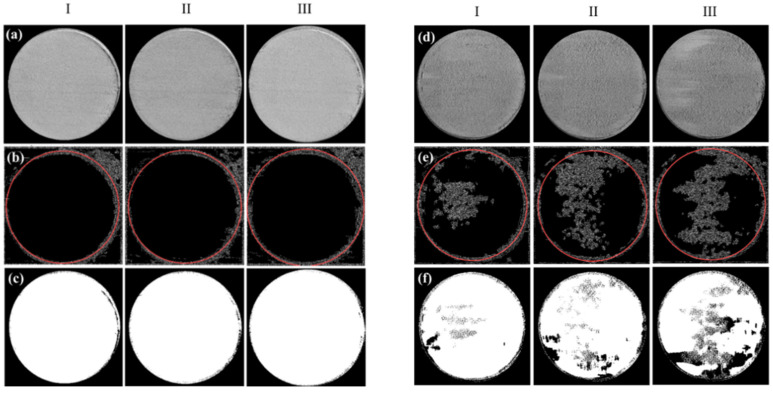
Cropped raw images of spread layers (30 mm circle diameter) of M15-45 (**a**-**I**) layer 1, (**a**-**II**) layer 2, and (**a**-**III**) layer 3. The same layers after edge detection (**b**-**I**) layer 1, (**b**-**II**) layer 2, and (**b**-**III**) layer 3. Followed by the application of threshold (**c**-**I**) layer 1, (**c**-**II**) layer 2, and (**c**-**III**) layer 3. Cropped raw images of spread layers of Mix1 (**d**-**I**) layer 1, (**d**-**II**) layer 2, and (**d**-**III**) layer 3. The same layers after edge detection (**e**-**I**) layer 1, (**e**-**II**) layer 2, and (**e**-**III**) layer 3. Followed by the application of threshold (**f**-**I**) layer 1, (**f**-**II**) layer 2, and (**f**-**III**) layer 3.

**Figure 7:**
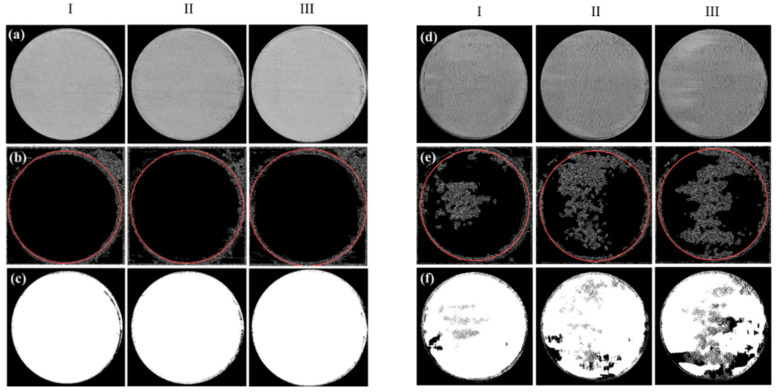
Cropped raw images of spread layers (30 mm circle diameter) of Mix2 (**a**-**I**) layer 1, (**a**-**II**) layer 2, and (**a**-**III**) layer 3. The same layers after edge detection (**b**-**I**) layer 1, (**b**-**II**) layer 2, and (**b**-**III**) layer 3. Followed by the application of threshold (**c**-**I**) layer 1, (**c**-**II**) layer 2, and (**c**-**III**) layer 3. Cropped raw images of spread layers of Mix3 (**d**-**I**) layer 1, (**d**-**II**) layer 2, and (**d**-**III**) layer 3. The same layers after edge detection (**e**-**I**) layer 1, (**e**-**II**) layer 2, and (**e**-**III**) layer 3. Followed by the application of threshold (**f**-**I**) layer 1, (**f**-**II**) layer 2, and (**f**-**III**) layer 3.

Spreadability was analyzed via MATLAB on the first three spread layers. Simply, in the beginning, the raw image taken was cropped as a circle, then edge detection and threshold were applied on the cropped circle. Followed by the determination of the black to white ratio of the threshold image which was used to identify the defects on the spread layer. The obtained and processed images, and the extracted data are given in Figure 6 and Figure 7 and Table 4, respectively. The latter demonstrates the average grayscale values and black to white ratios that are used to evaluate the spread quality of each layer.

As seen in Figure 6a–c, no irregularity (such as precipitates, clusters, waves) was observed from layers 1 to 3 of the spreads for M15-45. It was previously shown that M15-45 was a flowable powder (with a flow rate of 15.08 s/50 g and AOR value of 22.39°) and predictably homogeneous powder layers were observed. For M15-45 powder mixture layer 1, layer 2, layer 3, average grayscale values and black to white ratios were quantified as 119.84, 115.96, 135.05, and 0.31, 0.30, 0.21, respectively. 

The flow rate of Mix1 was not able to be assessed, and parallel to that, clear irregularities were observed from the raw and edge detection applied images for layers 1, 2, and 3. However, these irregularities were not noticed in threshold images (see Figure 6d–f). The average grayscale values of layers 1, 2, 3 were quantified as 94.49, 91.98, 92.66, while average black to white ratios were 0.37, 0.40, 0.46. It is important to note that Mix1 was a very cohesive powder with the highest AOR (32.85°) in all powder mixtures. Accordingly, different than all other samples an average grayscale value and black to the white ratio for all layers were quantified lower than 95 and higher than 0.32, respectively.

Mix2 was also a flowable powder blend akin to M15-45 but the flowability was higher (with a flow rate of 14.35 s/50 g and AOR value of 22.01°) due probably to coarser average particle size, compared to that of M15-45. While raw images demonstrate a homogeneous outlook (see Figure 7a), some extent of noise can be visible from the edge detection applied layer images (see Figure 7b) but not from the threshold applied ones given in Figure 7c. Accordingly, for Mix2 layer 1, 2, and 3, average grayscale values of 107.04, 107.38, 107.33, and black to white ratios of 0.31, 0.32, 0.31 were found, respectively.

The most flowable blend was the Mix3 with a flow rate of 14.19 s/50 g, and AOR being 21.33°. High flowability caused inadequate filling in layer 1, as shown in Figure 7(d-I). However, irregularities were not seen in the spread layers of 2 and 3, see Figure 7(d-II,III). The average grayscale values of layers 1, 2, and 3 were quantified as 82.38, 113.67, and 115.09, while average black to white ratios were found as 0.60, 0.30, and 0.30, respectively (see Figure 7e,f).

Although other factors such as spreading speed and blade type are highly possible to affect the spread layer characteristics [20], setting these constant while altering only the powder characteristics by changing the particle size distribution, it is possible to comment that for a homogenous layer covering, it is better to have a powder with average flowability value. Accordingly, for the tested powder blends the measured average grayscale value should not be lower than 95 on the cropped images, and black to a white ratio higher than 0.32 on threshold applied images. These results are in accordance with [27], where the spreadability was linked to the flowability by the AOR (lower AOR means higher flowability) and to powder specific energy (the lowest the specific energy the highest the flowability). 

### 3.3. Production of 3D Printed Cubes

**Figure 8:**
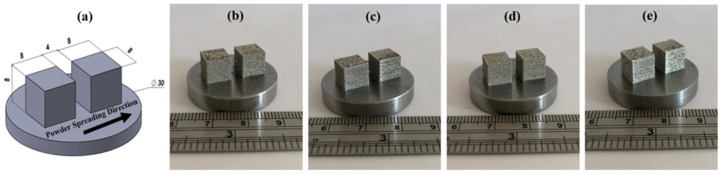
(**a**) 3D printing design showing the powder spreading direction, and PBF-SLM printed cubes by using the blends of (**b**) M15-45, (**c**) Mix1, (**d**) Mix2, and (**e**) Mix3.

To correlate the spreadability results with the quality of 3D printed parts, all blends were used to manufacture 3D-printed cubes. As seen in Figure 8, the printed samples have a surface finish which is generally seen in additively manufactured parts and might not be only due to particles with different PSD but other processing parameters [35]. 

As seen in the results reported in Table 5, all samples, including the M15-45 that was produced by using commercially recommended powder, show residual porosity. The calculated total porosity values were around 7 vol% from geometric, and 3–7 vol% from Archimedes technique, implying probable measurement errors. From Archimedes using also the assessed true density, it is possible to state that all samples contained both open and closed porosity for which the former remained ~2 vol% (see Table 5). 

**Figure 9:**
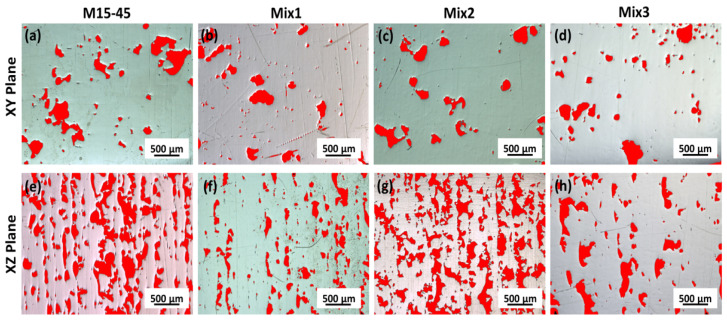
NIS-Elements BR image analysis processed optical microscope images taken from the top (XY plane: (**a**–**d**)) and left side (XZ plane: (**e**–**h**)) of the 3D printed cubes.

In addition, image processing was applied on three defined points on both top (XY plane) and left side (XZ plane) of the 3D printed cubes, see representative images given in Figure 9, and data in Table 6. It is clear from these analyses that for all produced samples a higher amount of surface porosity was observed on the XZ plane compared to that of the XY plane, being the XZ plane representative of a higher number of layers.

Among all samples, the lowest surface porosity together with high relative density (minimum total porosity) were obtained from the sample made by using Mix3 blend. This may be related to the layer thickness since during the production, layer thickness was always kept at 50 µm parallel to the D50 of Mix3, measured as ~50 µm. However, no other clear trend to observe the effects of spreadability on the final 3D printed parts was observed. It should be underlined that other SLM process parameters (laser power, speed, layer thickness, moisture, and oxygen level during the production, etc.) might also affect the final properties, for example, during the printing process spatter formation was noted and this probably altered the oxygen content, and the final pore formation [36,37,38], therefore certainly further studies with variations on the above-mentioned parameters are needed.

## 4. Conclusions

Powder spreadability was analyzed quantitatively via image processing algorithms using MATLAB. According to the applied algorithm on the tested powder blends, average grayscale values (0 to 255) lower than 95 on cropped images, and black to white ratio higher than 0.32 on threshold applied images, irregularities were observed. It was noted that when the powder mixture had a high flowability, it caused a reduction in the angle of repose (AOR), as seen for Mix3, demonstrating poor spreadability. In addition, when the volume fraction of fine particles (e.g., below 10 µm) increased, enhanced agglomeration decreased the flowability, and caused a similar poor spreadability effect. For example, Mix1 had the highest volume of particles sized below 10 µm (28.06%) and it demonstrated to have an average grayscale value lower than 95, and a black to a white ratio higher than 0.32. Therefore, it could be stated that when flowability is neither too high nor too low, but the one allowing an optimum coating, high-quality spreadability can be obtained. While the spread layer irregularities may cause unwanted microstructural developments, they can also be deliberately used to form porous components depending on the user’s requests.

3D printed samples were found to be porous which cannot be merely related to the poor spreadability but instead, is due probably to the combination of various factors including spreadability, spatter formation, scanning speed, laser speed, and other PBF-LB processing parameters [39,40]. As shown that it is currently not an easy task to investigate the correlation between spreadability and standardized characterization techniques. To propose a fully accepted method to quantify spreadability, there is still a need for more detailed and statistical studies. For example, while in the current work, spreadability was examined from the layers distributed at constant spreading speed (200 mm·s^−1^) with continuous layer thickness (50 µm); the alteration of these parameters and their impact with varying powder characteristics on the final component morphology together with further image enhancement techniques (e.g., contrast enhancement) will be conducted in future studies.

## Figures and Tables

**Table 1:** The processing parameters used in the PBF-LB (with Gaussian beam laser) for SLM.

Laser spot size (μm)	50
Layer thickness (μm)	50
Hatch distance (μm)	50
Laser power (W)	200
Laser wavelength (nm)	1064

**Table 2:** Particle size distribution (PSD) results of the samples measured with the laser diffraction method (data represent the average of three consistent measurements).

Sample Code	M15-45	Mix1	Mix2	Mix3
**D_10_ (µm)**	17.13	0.25	20.07	36.58
**D_50_ (µm)**	27.71	14.94	32.02	49.92
**D_90_ (µm)**	44.18	34.56	50.00	67.90
**≤10 (µm)**	0	28.06	0	0
**≤20 µm (%)**	19.66	66.58	9.82	0
**20 µm–38 µm (%)**	60.37	26.13	58.21	13
**38 µm–45µm (%)**	10.91	3.78	14.98	20.42
**45 µm–53 µm (%)**	6.17	2.40	9.97	26.34
**53 µm–63 µm (%)**	2.65	1.11	5.55	23.41
**63 µm–75 µm (%)**	0.24	0	1.47	13.02
**75 µm–90 µm (%)**	0	0	0	3.56
**90 µm–106 µm (%)**	0	0	0	0.25
**106 µm–125 µm (%)**	0	0	0	0

**Table 3:** Density, flow rate, AOR results (average of five consistent measurements), together with oxygen content measured by hot gas extraction, for M15-45, Mix1, Mix2, and Mix3.

Sample Code	M15-45	Mix1	Mix2	Mix3
**Apparent density (g cm^−3^)**	4.78	4.97	4.80	4.86
**Tap density (g cm^−3^)**	5.29	5.55	4.96	5.23
**Flow rate (s/50 g)**	15.08	-	14.35	14.19
**Angle of Repose (AOR, °)**	22.39	32.85	22.01	21.33
**Oxygen amount (ppm)**	246	384	241	207

**Table 4:** The average grayscale values (calculated from the cropped raw image) and black to white ratios (calculated from threshold image) of each layer.

Sample	Average Grayscale Value (0 to 255)	Black to White Ratio
**M15-45-Layer 1**	119.84	0.31
**M15-45-Layer 2**	115.96	0.30
**M15-45-Layer 3**	135.05	0.26
**Mix1-Layer 1**	94.49	0.37
**Mix1-Layer 2**	91.98	0.40
**Mix1-Layer 3**	92.66	0.46
**Mix2-Layer 1**	107.04	0.31
**Mix2-Layer 2**	107.38	0.32
**Mix2-Layer 3**	107.33	0.31
**Mix3-Layer 1**	82.38	0.60
**Mix3-Layer 2**	113.67	0.30
**Mix3-Layer 3**	115.09	0.30

**Table 5:** Bulk density (g·cm^−3^) and porosity (%) measurement results (mean ave. ± standard deviation) of the cubes. True density was measured from the powder as 8.64 ± 0.009 g cm^−3^ by using He pycnometer.

Sample	Bulk Density (g·cm^−3^) (Geometric)	Total Porosity (vol%) (Geometric)	Bulk Density (g·cm^−3^) (Archimedes)	Total Porosity (vol%) (Archimedes)	Open Porosity (vol%) (Archimedes)
**3DM15-45**	8.09 ± 0.005	6.34 ± 0.057	8.37 ± 0.037	3.07 ±0.435	1.51
**3DMix1**	8.05 ± 0.030	6.80 ± 0.353	8.15 ± 0.002	5.71 ± 0.028	2.47
**3DMix2**	8.03 ± 0.012	6.99 ± 0.139	8.07 ± 0.032	6.58 ± 0.369	2.03
**3DMix3**	8.05 ± 0.033	6.87 ± 0.379	8.65 ± 0.418	3.52 ± 1.180	1.67

**Table 6:** Image processing data giving the surface porosity (%) data (mean ave. ± standard deviation) obtained from 3 different points on two different 3D printed cubes of the same set.

Sample	Porosity (%) on XY Plane	Sample	Porosity (%) on XZ Plane
**3DM15-45-XY**	7.04 ± 3.22	**M15-45-XZ**	13.50 ± 8.25
**3DMix1-XY**	5.53 ± 3.06	**Mix1-XZ**	8.18 ± 2.06
**3DMix2-XY**	4.23 ± 0.97	**Mix2-XZ**	18.98 ± 4.88
**3DMix3-XY**	5.57 ± 3.29	**Mix3-XZ**	6.72 ± 3.23

## Data Availability

The data presented in this study are available upon request from the corresponding author.
